# Disruptive and complementary effects of depression symptoms on spontaneous brain activity in the subcortical vascular mild cognitive impairment

**DOI:** 10.3389/fnagi.2024.1338179

**Published:** 2024-09-17

**Authors:** Liyu Hu, Jianxiang Chen, Xinbei Li, Haoran Zhang, Jinhuan Zhang, Yingqi Lu, Jie Lian, Haibo Yu, Nan Yang, Jianjun Wang, Hanqing Lyu, Jinping Xu

**Affiliations:** ^1^Department of Acupuncture and Moxibustion, Shenzhen Traditional Chinese Medicine Hospital, The Fourth Clinical Medical College, Guangzhou University of Chinese Medicine, Shenzhen, China; ^2^Institute of Biomedical and Health Engineering, Shenzhen Institutes of Advanced Technology, Chinese Academy of Sciences, Shenzhen, China; ^3^Department of Radiology, Shenzhen Traditional Chinese Medicine Hospital, The Fourth Clinical Medical College, Guangzhou University of Chinese Medicine, Shenzhen, China; ^4^Department of Neurology and Psychiatry, Shenzhen Traditional Chinese Medicine Hospital, The Fourth Clinical Medical College, Guangzhou University of Chinese Medicine, Shenzhen, China, 5Hospital of Traditional Chinese Medicine of Zhongshan, Shenzhen, China; ^5^Hospital of Traditional Chinese Medicine of Zhongshan, Zhongshan, China

**Keywords:** subcortical vascular mild cognitive impairment, depression symptoms, fractions of amplitude of low-frequency fluctuations, resting-state functional connectivity, support vector machine

## Abstract

**Background:**

Although depression symptoms are commonly reported in patients with subcortical vascular mild cognitive impairment (svMCI), their impact on brain functions remains largely unknown, with diagnoses mainly dependent on behavioral assessments.

**Methods:**

In this study, we analyzed resting-state fMRI data from a cohort of 34 svMCI patients, comprising 18 patients with depression symptoms (svMCI+D) and 16 patients without (svMCI-D), along with 34 normal controls (NC). The study used the fraction of the amplitude of low-frequency fluctuations (fALFF), resting-state functional connectivity, correlation analyses, and support vector machine (SVM) techniques.

**Results:**

The fALFF of the right cerebellum (CERE.R) differed among the svMCI+D, svMCI-D, and NC groups. Specifically, the regional mean fALFF of CERE. R was lower in svMCI-D patients compared to NC but higher in svMCI+D patients compared to svMCI-D patients. Moreover, the adjusted fALFF of CERE. R showed a significant correlation with Montreal Cognitive Assessment (MOCA) scores in svMCI-D patients. The fALFF of the right orbital part of the superior frontal gyrus was significantly correlated with Hamilton Depression Scale scores in svMCI+D patients, whereas the fALFF of the right postcingulate cortex (PCC.R) showed a significant correlation with MOCA scores in svMCI-D patients. Furthermore, RSFC between PCC. R and right precuneus, as well as between CERE. R and the right lingual gyrus (LING.R), was significantly reduced in svMCI-D patients compared to NC. In regional analyses, the adjusted RSFC between PCC. R and PreCUN. R, as well as between CERE. R and LING. R, was decreased in svMCI-D patients compared to NC but increased in svMCI+D patients compared to svMCI-D. Further SVM analyses achieved good performances, with an area under the curve (AUC) of 0.82 for classifying svMCI+D, svMCI-D, and NC; 0.96 for classifying svMCI+D and svMCI-D; 0.82 for classifying svMCI+D and NC; and 0.92 for classifying svMCI-D and NC.

**Conclusion:**

The study revealed disruptive effects of cognitive impairment, along with both disruptive and complementary effects of depression symptoms on spontaneous brain activity in svMCI. Moreover, these findings suggest that the identified features might serve as potential biomarkers for distinguishing between svMCI+D, svMCI-D, and NC, thereby guiding clinical treatments such as transcranial magnetic stimulation for svMCI.

## Introduction

Mild cognitive impairment (MCI) has been considered a preclinical stage of dementia or Alzheimer’s disease (AD) (Lyketsos et al., 2002). In addition to cognitive impairment, neuropsychiatric symptoms (Donaghy et al., 2018; Lo et al., 2020; Gallagher et al., 2017), especially depression, have been commonly observed in MCI, as well as in frontotemporal dementia and Lewy-body disease, with an overall pooled prevalence of 32% (Ismail et al., 2017; Cotta et al., 2021).

Moreover, several meta-analyses have consistently shown that depression is associated with a higher likelihood of progression from MCI to AD (Ismail et al., 2017; Gao et al., 2013; Cooper et al., 2015; Mourao et al., 2016). As one of the common subtypes of MCI, subcortical vascular mild cognitive impairment (svMCI) is clinically characterized by several neuroimaging features, including white matter hyperintensities, lacunar infarction, micro-infarcts, micro-bleeds, enlarged perivascular spaces, and brain atrophy. Among them, vascular lesions in the deep and periventricular white matter and in the deep gray matter, as well as a greater number of perivascular spaces in the basal ganglia, are particularly implicated in cognitive decline, potentially leading to vascular dementia (Seo et al., 2009; Lee et al., 2018; Román et al., 2002; Ramusino et al., 2022; Wardlaw et al., 2013). Similar to MCI, depression in svMCI has been reported not only as a predictive marker for conversion to subcortical vascular dementia (SvaD) (Kim et al., 2013) but also as a factor that exacerbates cognitive impairments (Steffens, 2012; Cinar et al., 2014).

Given that depression in svMCI may be reversible through the management of risk factors (Ravaglia et al., 2005; Jak et al., 2009), exploring the underlying neurobiological mechanisms of depression in the svMCI is crucial for preventing the progression from svMCI to SvaD.

The amplitude of low-frequency fluctuation (ALFF) and fractional ALFF (fALFF) (Zou et al., 2008) have been widely and effectively adopted to explore changing patterns in spontaneous brain activity in the low-frequency range in patients with MCI (Han et al., 2011; Han et al., 2012; Zhao et al., 2015; Yang et al., 2018; Wang et al., 2019) and svMCI (Yi et al., 2012). These measures have proven effective in characterizing early and gradual physiological changes associated with AD (Yang et al., 2018). However, these studies showed inconsistent results, showing both increased and/or decreased activity patterns in different brain regions. The potential effect of co-existing neuropsychiatric symptoms, particularly depression, on brain function is an important factor that must not be overlooked.

Recently, there has been growing interest in understanding how depression symptoms affect brain activity in MCI (Li et al., 2017; Liu et al., 2018; Yu et al., 2019). For example, one study showed significant differences in fALFF across the full frequency, slow-5, and slow-4 bands in MCI patients with depression (Li et al., 2017). Another study identified decreased fALFF in regions such as the temporal gyrus, frontal gyrus, inferior occipital gyrus, middle frontal gyrus, and cerebellum, alongside increases in the cuneus, calcarine, and lingual gyrus in MCI with depression (Yu et al., 2019).

To the best of our knowledge, no studies to date have explored the impact of depression symptoms on spontaneous brain activity in svMCI.

In the current study, we aimed to explore (1) whether and how depression symptoms affect spontaneous brain activity in patients with svMCI and (2) whether these altered neural indices can serve as biomarkers for diagnosing svMCI. To address these two questions, resting-state fMRI data were obtained from 34 svMCI patients, comprising 18 svMCI patients with depression symptoms (svMCI+D), 16 svMCI patients without depression symptoms (svMCI-D), and 34 normal controls (NC).

**Figure 1:**
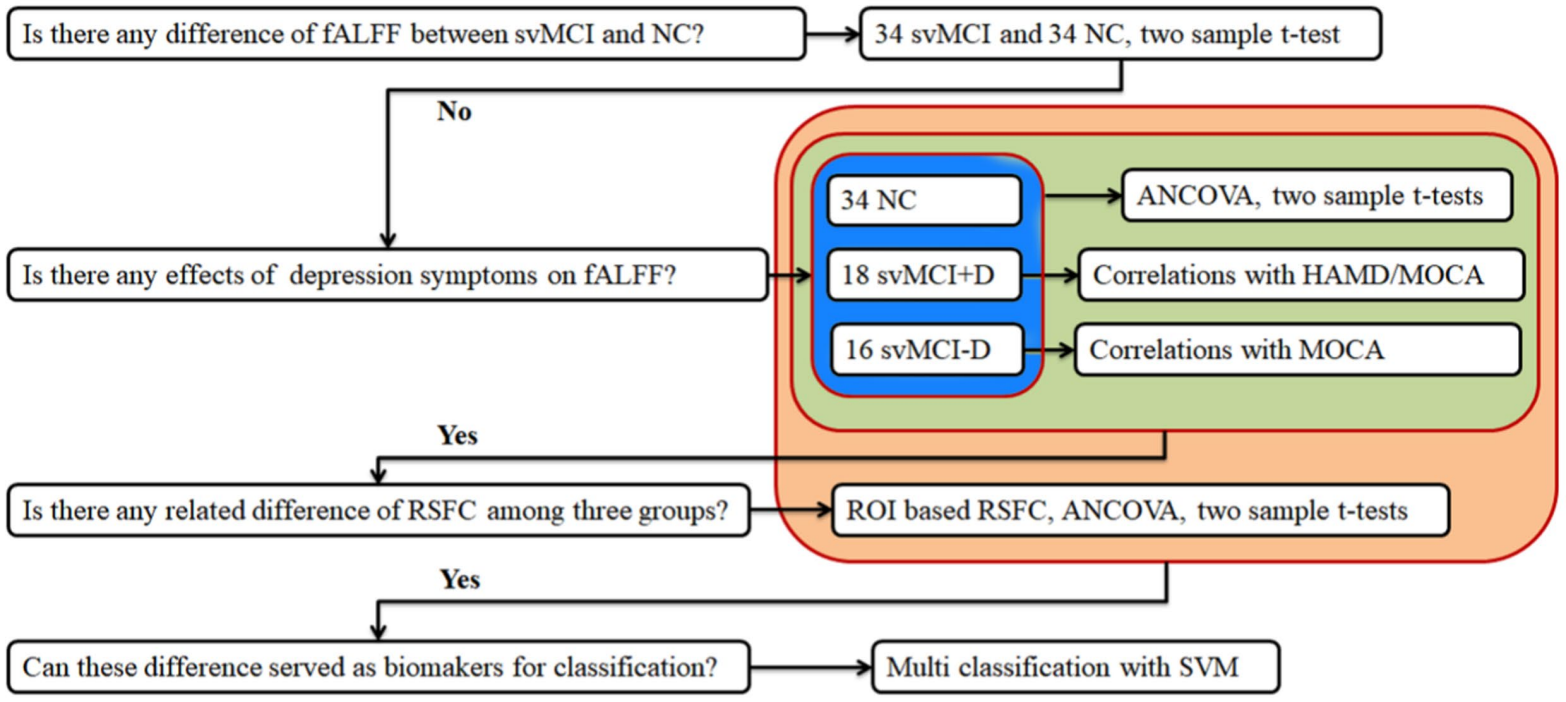
Flowchart of study design. ALFF, amplitude of low-frequency fluctuations, fALFF, fractions of ALFF; svMCI, subcortical vascular mild cognitive impairment; NC, normal controls; svMCI+D, svMCI with depression symptoms; svMCI-D patients, svMCI patients without depression symptoms; HAMD, Hamilton Depression Scale; MOCA, Montreal Cognitive Assessment; ANCOVA, analysis of covariance; RSFC, resting state functional connectivity; and SVM, support vector machine.

Then, several main steps were performed in the study (Figure 1): (1) comparisons of ALFF and fALFF between svMCI and NC groups; (2) comparisons of ALFF and fALFF among svMCI-D, svMCI-D, and NC groups; (3) correlation analyses between ALFF/fALFF and Hamilton Depression Scale (HAMD)/Montreal Cognitive Assessment (MOCA) scores in the svMCI+D group, as well as between ALFF/fALFF and MOCA scores in the svMCI-D group; (4) analysis of resting-state functional connectivity (RSFC) to identify related spontaneous brain functional patterns; and (5) multivariate pattern analyses using a linear support vector machine (SVM) to determine whether these altered neural indices can serve as biomarkers for diagnosing svMCI.

## Methods

### Participants

This study was conducted at the Shenzhen Traditional Chinese Medicine Hospital and approved by the Institutional Review Board. Patients and normal controls (NCs) were recruited through advertisements at the hospital, and all participants provided written informed consent. The svMCI patients were required to meet specific diagnostic, exclusion, and MRI criteria, whereas NC participants only needed to meet the exclusion criteria.

The diagnostic criteria for svMCI patients included: (1) reported cognitive impairments involving memory and/or other cognitive domains lasting for at least 3 months; (2) not classified as normal nor demented, with a clinical dementia rating of (CDR) > = 0.5 in at least one domain (Hughes et al., 1982) and a global score of <= 0.5 in the three functional CDR domains (Salloway et al., 2004); and (3) a MOCA score of <= 26.

The exclusion criteria were as follows: (1) history of psychiatric illness in any first-to third-degree biological relatives; (2) history of strokes or transient ischemic attack within the past 3 months; (3) history of seizures, schizophrenia, or major depressive disorder; (4) inherited or inflammatory small vessel disease; (5) previous head trauma with the loss of consciousness; (6) use of medications (e.g., Donepezil and Kabalatine) that may affect cognition; (7) serious medical or surgical illness; (8) physical disabilities that could prevent completion of neuropsychological testing; and (9) contraindications for MRI.

The MRI criterion was based on T2 FLAIR images, and patients needed to meet at least one of the following: (1) at least three supratentorial subcortical small infarcts (diameters ranged from 3 to 20 mm) with or without white matter lesions of any degree; (2) moderate to severe white matter lesions (a score of > = 2 according to the Fazekas rating scale (FRS)) (Fazekas et al., 1987) with/without small infarct; (3) one or more strategically located subcortical small infarcts in the caudate nucleus, globus pallidus, or thalamus.

The severity of depression symptoms in patients was assessed using the 17-item HAMD. Patients were then categorized into two groups: svMCI+D (HAMD score 8–17) and svMCI-D (HAMD score <7) (Zimmerman et al., 2013). Ultimately, 34 svMCI patients (18 svMCI+D and 16 svMCI-D) and 34 NC were included in this study (Table 1).

**Table 1:** Demographic data and clinical measures.

Groups	svMCI	NC	*T*-value	*p*-value
Subjects	34	34	–	–
Age (mean ± SD)	63.32 ± 6.81	61.4 ± 4.96	1.505	0.137^a^
Gender (men/women)	16/18	14/20	–	0.807^b^
Education (mean ± SD)	8.29 ± 3.77	9.94 ± 3.66	−1.826	0.072^a^
MOCA (mean ± SD)	19.50 ± 2.14	27.91 ± 1.04^c^	−17.40	<0.001^a^
HAMD (mean ± SD)	8.32 ± 3.86	2.30 ± 1.55^c^	7.074	<0.001^a^

Groups	svMCI+D	svMCI-D	NC	*F*-value	*p*-value
Subjects	18	16	34	–	–
Age (mean ± SD)	61.66 ± 6.49	65.18 ± 6.88	61.4 ± 4.96	2.689	0.076^d^
Sex (male/female)	8/10	8/8	14/20	–	0.842^a^
Education (mean ± SD)	7.66 ± 3.77	9.00 ± 3.75	9.94 ± 3.66	2.214	0.117^d^
MOCA (mean ± SD)	18.83 ± 1.68	20.25 ± 2.40	27.91 ± 1.04^c^	167.43	<0.001^d^
HAMD (mean ± SD)	11.38 ± 2.50	4.87 ± 1.36	2.30 ± 1.55^c^	123.73	<0.001^d^

^a^Represents two-sample *t*-tests; ^b^represents *χ*^2^; ^c^only data of 24 people were available; and ^d^represents an analysis of variance. svMCI, subcortical vascular mild cognitive impairment; svMCI + D, svMCI with depression; svMCI-D, svMCI without depression; NC, normal controls, SD, standard deviation, MOCA, Montreal Cognitive Assessment; and HAMD, Hamilton Depression Scale.

### MRI acquisition

MRI data of all participants were acquired using a GE medical system 3 T scanner (MR750) in the radiology department. The fMRI images were obtained with the following parameters: repetition time (TR) = 2000 ms, echo time (TE) = 35 ms, flip angle = 90 degrees, matrix size = 64 × 64, slice thickness = 4 mm, voxel size = 4 × 4 × 4 mm^3^, field of view = 220 × 220 mm^2^, and volumes = 240. The parameters for T2 FLAIR images were as follows: TR = 9,000 ms, TE = 92.544 ms, flip angle = 160 degrees, matrix size = 256 × 224, slice thickness = 6.5 mm, and voxel size = 0.47 × 0.47 × 6.5 mm^3^.

### Image preprocessing

The resting-state fMRI data were preprocessed using DPABI^1^ following these steps: (1) removal of the first 10 volumes; (2) slice timing correction; (3) realignment (subjects with head motion exceeding 3 mm in any dimension or 3° of angular motion were removed, leading to the removal of one svMCI-D patient); (4) spatial normalization using an echo planar imaging (EPI) template; (5) resampling to 3 mm isotropic voxels; (6) smoothing with a Gaussian kernel of 6 mm full-width at half maximum; (7) regressing of linear and quadratic trends, signals of 24 head motion parameters (Satterthwaite et al., 2013; Yan et al., 2013), as well as signals from the white matter and cerebrospinal fluid. For the RSFC analysis, additional three steps were performed: (1) regressing out global signals; (2) temporal band-pass filtering (0.01–0.1 Hz); and (3) “scrubbing” two-time points before and one-time points after bad images, with a frame displacement (FD) of >0.5 (Power et al., 2012).

### Calculations of ALFF, fALFF, and RSFC

Following preprocessing, voxel-wise ALFF and fALFF were calculated using DPABI. Similar to previous studies (Zou et al., 2008; Yang et al., 2007), the ALFF was defined as the mean square root of the power spectrum of time series within the 0.01–0.1 Hz frequency range, whereas fALFF was calculated as the ratio of the power spectrum of time series within the 0.01–0.01 Hz range to that of the entire frequency range. These values were further normalized using Fisher’s z transformation to improve normality.

For altered brain regions, RSFC of the whole brain was further calculated to investigate functional changing patterns. First, Pearson correlation coefficients were calculated between the mean time series of each altered brain region and that of each voxel in the rest of the brain. Then, each correlation coefficient was converted to z values using Fisher’s z transformation to improve normality.

### Statistical analysis

Several main statistical steps were taken using DPABI: (1) two sample *t*-tests to examine differences in the voxel-wise ALFF and fALFF maps between svMCI and NC with age, sex, and education as covariates; (2) one-way analysis of covariance (ANCOVA) and *post-hoc* two-sample *t*-tests between any two groups to examine between-group differences in the voxel-wise ALFF, fALFF and RSFC maps among scMCI+D, svMCI-D, and NC patients with age, sex, and education as covariates; (3) correlation analyses between HAMD scores and voxel-wise ALFF and fALFF maps, with age, sex, education, and MOCA scores as covariates in the svMCI+D; (4) correlation analyses between MOCA scores and voxel-wise ALFF and fALFF maps, with age, sex, education, and HAMD scores as covariates in the svMCI+D; (5) correlation analyses between MOCA scores and voxel-wise ALFF and fALFF maps, with age, sex, education, and HAMD scores as covariates in the svMCI+D. All these statistical analyses were corrected using the Gaussian Random Field (GRF) corrections, with a voxel level of *p* < 0.001 and a cluster level of *p* < 0.05.

In addition, we calculated the regional mean fALFF and RSFC for the regions that showed significant differences among the three groups. Group differences were compared among three groups using ANCOVA and *post-hoc t*-tests between any two groups, with age, sex, and education as covariates, using IBM SPSS 19. We also conducted correlation analyses between regional mean fALFF and clinical measures for the svMCI+D and svMCI-D groups, respectively, using IBM SPSS 19. For the svMCI+D patients, age, sex, education, and MOCA scores were regressed out from the fALFF, whereas for the svMCI-D patients, age, sex, education, and HAMD scores were regressed out from the fALFF. The resulting values were defined as adjusted fALFF, following the same adjustment method applied to the RSFC. The significance level was set at *p <* 0.05.

### Classifications using a support vector machine

To determine whether the identified neural indices might serve as biomarkers for the classification of svMCI+D, svMCI-D, and NC, we employed a linear support vector machine (SVM) using the LIBSVMs toolkit (Chang and Lin, 2011). The features used for classification include the mean regional fALFF of the right orbitofrontal cortex (OFC.R), right posterior cingulate cortex (PCC.R), and right cerebellum (CERE.R), as well as the functional connectivity between PCC. R and the right precuneus (PreCUN.R), and between CERE. R and the right lingual gyrus (LING.R). We conducted classifications among svMCI+D, svMCI-D, and NC, as well as between svMCI+D and svMCI-D, between svMCI+D and NC, and between svMCI-D and NC. Given the relatively small sample size, we used a leave-one-out cross-validation strategy to estimate the generalization ability of our classifiers. The performance of classifiers was assessed using the area under curve (AUC).

## Results

### Demographic data and clinical measures

The normality of the distribution was analyzed using the Shapiro–Wilk test. The education years, MOCA scores, and HAMD scores were all normally distributed. Therefore, demographic and clinical measures between the two groups were analyzed using two-sample *t*-tests, whereas the three groups were analyzed using one-way ANOVA. Sex was analyzed using the chi-square (*χ*^2^) test. As a result, no significant difference was found in age, sex, or education, whereas significant differences were found in MOCA and HAMD scores between svMCI and NC, among svMCI+D, svMCI-D, and NC (Table 1).

### Group differences between ALFF and fALFF

**Figure 2:**
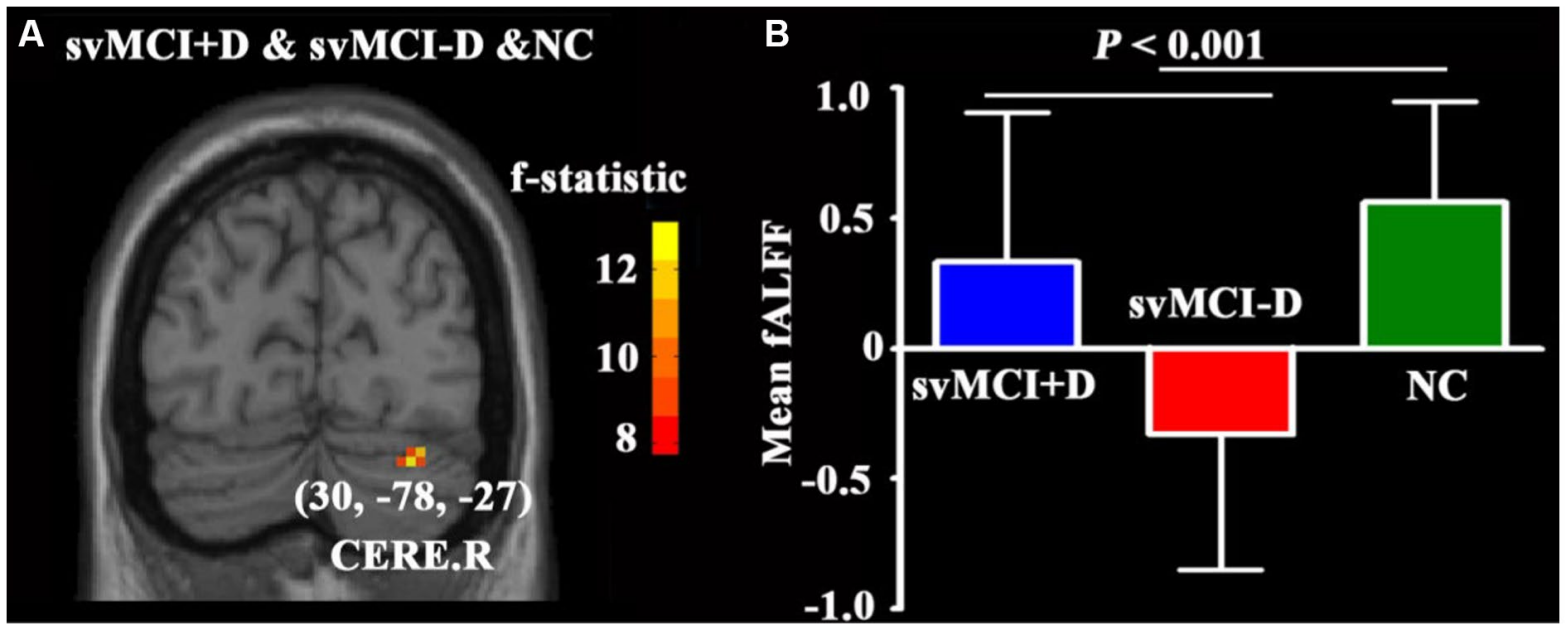
**(A)** ANCOVA of fALFF among svMCI+D, svMCI-D, and NC using DPABI with age, sex, and education as covariates. The results were corrected by Gaussian Random Field (GRF) with a voxel level *p* < 0.001 and a cluster level *p* < 0.05. **(B)** ANCOVA and *post-hoc* two-sample *t*-tests of mean fALFF were performed using SPSS.

No significant differences in ALFF and fALFF were observed between svMCI patients and NC. However, the fALFF of the CERE. R showed significant alterations among the svMCI+D, svMCI-D, and NC patients (Figure 2A). Specifically, the regional mean fALFF of CERE. R was decreased in svMCI-D patients compared to NC, but it increased in svMCI+D patients compared to svMCI-D patients (Figure 2B).

### Correlation results

**Figure 3:**
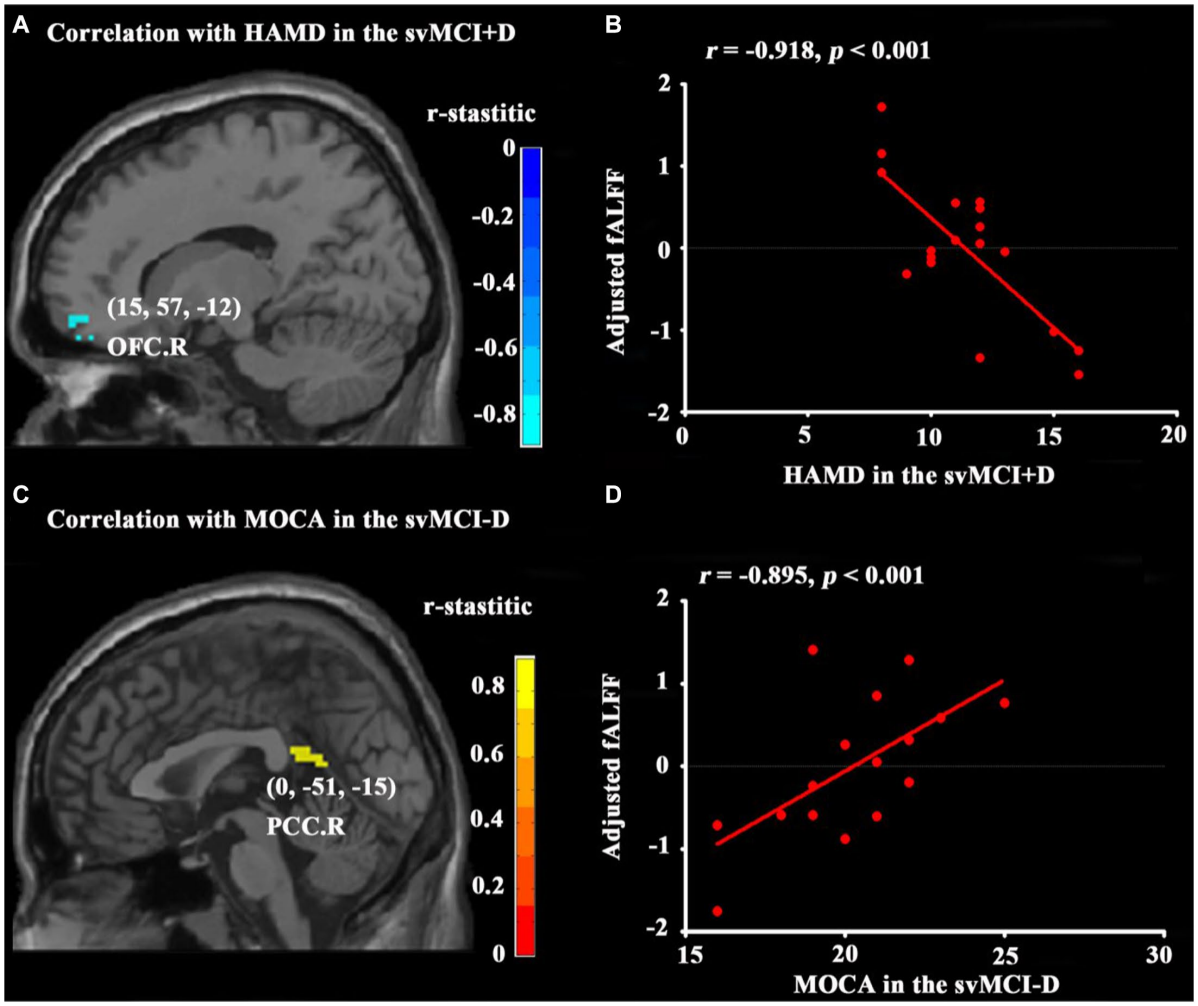
Results of correlation analyses. Correlation analyses between HAMD and whole brain fALFF in svMCI+D patients **(A)** with age, sex, education, and MOCA as covariates and between MOCA and whole brain fALFF in the svMCI-D **(C)** with age, sex, education, and HAMD as covariates were obtained using DPABI (see text footnote 1). The results were corrected by GRF with a voxel level *p* < 0.001 and a cluster level *p* < 0.05. Correlation analyses between HAMD and adjusted fALFF regressed by age, sex, education, and MOCA in the svMCI+D **(B)** and between MOCA and adjusted fALFF regressed by age, sex, education, and HAMD in the svMCI-D **(D)** were obtained using SPSS. The significant level was set at *p* < 0.05.

No significant correlations were found between ALFF and clinical measurements in either the svMCI+D or svMCI-D patients. However, the fALFF of the OFC. R was negatively correlated with HAMD scores in svMCI+D patients after controlling for age, sex, education, and MOCA scores (Figures 3A,B). Additionally, the fALFF of the PCC. R was negatively correlated with MOCA scores in svMCI-D patients while controlling for age, sex, education, and HAMD scores (Figures 3C,D).

### Group differences of RSFC

**Figure 4:**
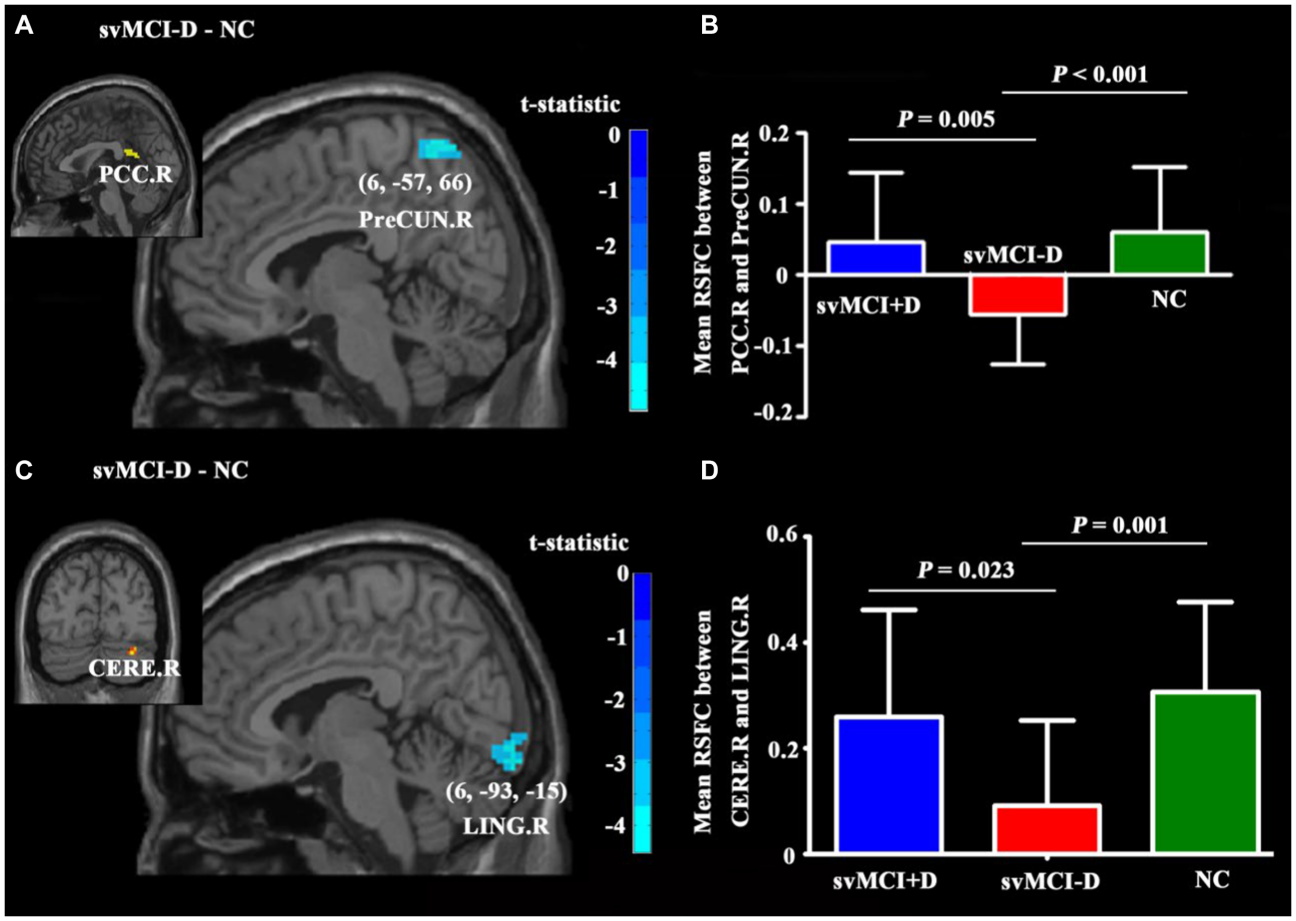
Differences in RSFC among the three groups. **(A)** The PreCUN.R showed significantly decreased RSFC with PCC. R in the svMCI-D compared to NC. **(B)** The mean RSFC between PCC. R and PreCUN. R was the lowest in the svMCI-D. **(C)** The LING. R showed significantly decreased RSFC with CERE. R in the svMCI-D compared to NC. **(D)** The mean RSFC between CERE. R and LING. R was the lowest in the svMCI-D. Abbreviations: PreCUN. R, the right precuneus; PCC. R, the right postcingulate cortex; CERE. R, the right cerebellum; and LINGnnn R, the right lingual gyrus.

In voxel-wise analyses, RSFC between PCC. R and PreCUN. R, as well as between CERE. R and LING. R, was significantly decreased in svMCI-D patients compared to NC (Figures 4A,C). In regional analyses, the adjusted RSFC between PCC. R and PreCUN. R, as well as between CERE. R and LING. R, was decreased in svMCI-D patients compared to NC but increased in svMCI+D patients compared to svMCI-D patients (Figures 4B,D).

### SVM results

**Figure 5:**
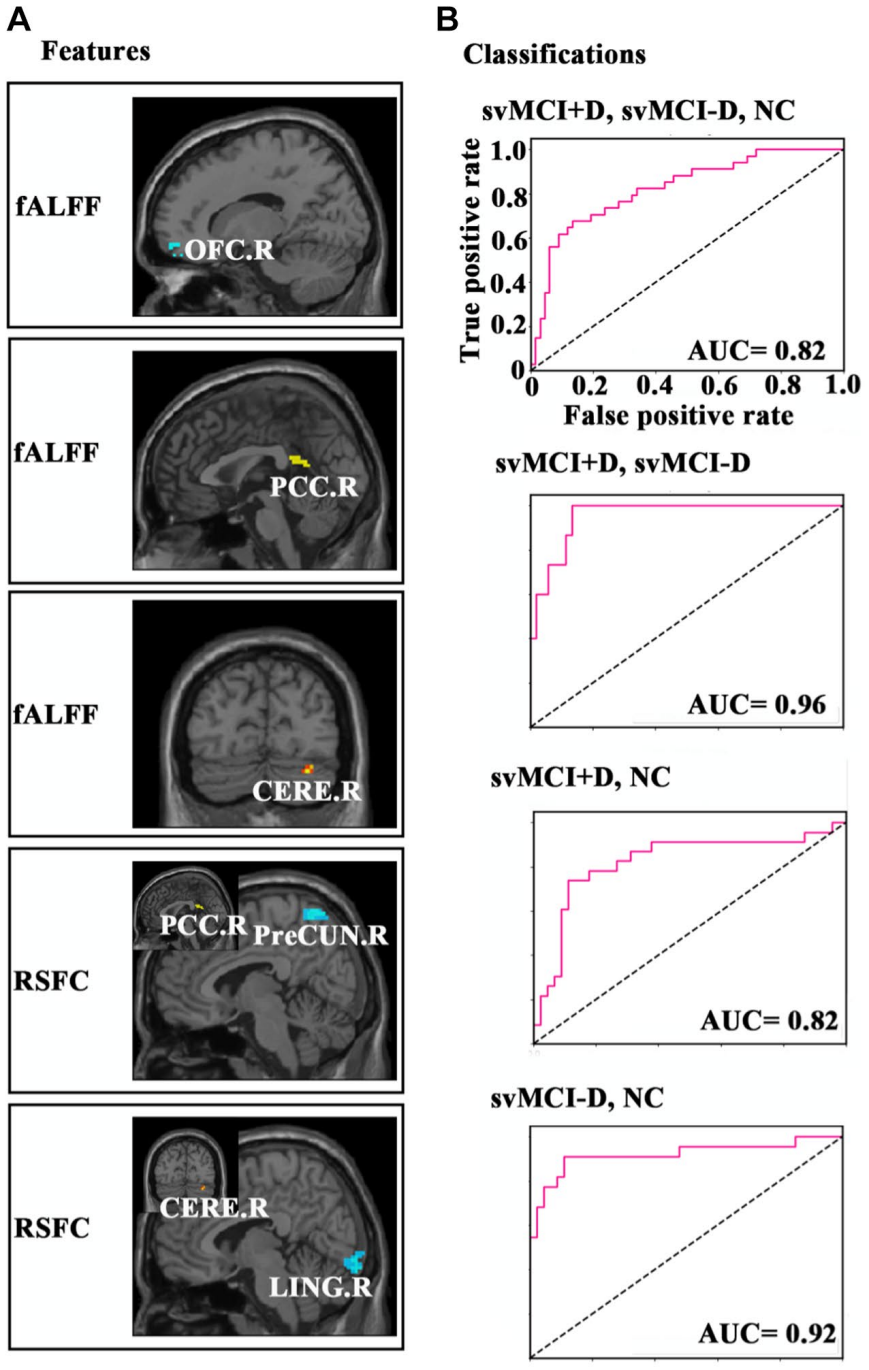
**(A)** Features of the SVM. **(B)** ROC of the classification results.

The SVM classifier demonstrated strong performance, achieving an AUC of 0.82 for distinguishing among svMCI+D, svMCI-D, and NC; an AUC of 0.96 for distinguishing between svMCI+D and svMCI-D patients; an AUC of 0.82 for distinguishing between svMCI+D and NC; and an AUC of 0.92 for distinguishing between svMCI-D and NC (Figure 5).

## Discussion

In the present study, we conducted ALFF, fALFF, RSFC, and correlation analyses to investigate the impact of depression symptoms on spontaneous brain activity in svMCI patients and used SVM to assess whether the identified neural indices might serve as biomarkers for classifying svMCI+D, svMCI-D, and NC patients. Four main results emerged: (1) fALFF in CERE. R, RSFC between CERE. R and LING. R, and RSFC between PCC. R and PreCUN. R were decreased in svMCI-D patients compared to NC but increased in svMCI+D patients compared to svMCI-D patients; (2) fALFF in OFC. R was significantly correlated with depression symptoms in svMCI+D patients; (3) fALFF in PCC. R was significantly correlated with cognitive impairment in svMCI-D patients; and (4) further SVM analyses achieved good performances, with an AUC of 0.96 for distinguishing svMCI+D patients from svMCI-D patients.

First, we observed that the fALFF of CERE. R decreased in svMCI-D patients compared to NC but increased in svMCI+D patients compared to svMCI-D patients. Although the CERE is widely known to play an important role in regulating motor coordination, accumulating evidence has highlighted its significant involvement in cognitive regulation (Baillieux et al., 2008) and emotion processing (Stoodley and Schmahmann, 2009). Indeed, functional abnormalities in the CERE have been reported not only in patients with depression (Liu et al., 2013; Guo et al., 2015; Xu et al., 2017) but also across all various subtypes of MCI, including aMCI (Wang et al., 2016; Lin et al., 2020), vascular mild cognitive impairment (VaMCI) (Zuo et al., 2019; Diciotti et al., 2017), and svMCI (Seo et al., 2009; Yoon et al., 2013). Recent studies on MCI with depression have also noted decreased fALFF and greater loss of gray matter volume in the CERE (Yu et al., 2019; Capogna et al., 2019). In addition, two of our previous studies showed similar patterns of RSFC and white matter microstructure in the CERE. R in svMCI+D patients (Lyu et al., 2019; Xu et al., 2020).

The decreased brain activity in svMCI-D patients may be linked to the detrimental effects of cognitive impairment. However, the increased brain activity observed in svMCI+D patients could suggest compensatory effects related to depression symptoms, although we found no significant correlation between these alterations and HAMD scores. This lack of correlation may be due to the relatively small sample size, and caution is warranted when interpreting these findings. Nonetheless, identifying increased brain activity in svMCI+D patients is valuable for a better understanding of the underlying mechanism of depression in svMCI patients.

Then, we also found that the fALFF of the OFC. R was negatively correlated with HAMD scores in svMCI+D patients. The OFC is believed to be involved in the non-reward attractor theory of depression, a concept supported by many neurophysiological, functional neuroimaging, and lesion studies. Additionally, the OFC is a key component of the frontal-limbic circuit, which is closely associated with depressive symptoms (Rolls, 2016; Cotta Ramusino et al., 2024). Many previous studies showed decreased gray matter volume in the OFC among patients with depression (Lavretsky et al., 2005; Salvadore et al., 2011; Grieve et al., 2013; Kandilarova et al., 2019; Harada et al., 2016). Moreover, the efficiency of the right orbital part of the superior frontal gyrus (part of OFC) was also found to be significantly correlated with the severity of depression (Qin et al., 2014). Similarly, another study showed that depressive symptoms in MCI are associated with gray matter volume loss in the OFC (Xie et al., 2012). This body of evidence supports our finding that the decreased fALFF in the OFC. R is strongly associated with the detrimental effects of depression symptoms in svMCI+D patients.

In addition, we found that the fALFF of the PCC. R was negatively correlated with MOCA scores, suggesting that cognitive impairments in svMCI-D patients may have damaging effects. As a key region in the default mode network (Davey et al., 2016; Satpute and Lindquist, 2019), the PCC plays an important role in various cognitive functions, such as episodic memory, spatial attention, and self-evaluation (Davey et al., 2016; Bagurdes et al., 2008; Natu et al., 2019). Moreover, numerous histopathological (Pivtoraiko et al., 2015; Mitolo et al., 2019), metabolic (Johnson et al., 2006; Frings et al., 2010; Gusnard et al., 2001), structural (Zhao et al., 2015; Choo et al., 2010), and functional imaging (Wang et al., 2016; Krajcovicova et al., 2017; Teipel et al., 2017; Papma et al., 2017) studies have consistently shown that the PCC is highly associated with the pathophysiology of MCI and may even serve as an imaging predictor for further cognitive decline into clinical AD (Huang et al., 2002; Petrella et al., 2007; Zhou et al., 2008). A three-year longitudinal analysis also found that altered functional connectivity of the PCC was significantly correlated with cognitive performances in MCI (Wang et al., 2012). Although these results primarily focus on MCI, they are similar to our results, providing additional evidence that alterations in the PCC may be linked to cognitive impairment in svMCI patients.

To the best of our knowledge, only three previous studies have reported functional alterations of the PCC in svMCI patients. First, Sun et al. (2011) identified abnormal functional connectivity of the PCC using resting-state fMRI in patients with vascular cognitive impairment, no dementia (VCIND), and a clinical state with a vascular etiology similar to that of svMCI. Subsequently, Yi et al. (2012) observed significantly increased low-frequency oscillation amplitudes in the PCC in svMCI patients. Finally, significantly decreased rsFC of the PCC and its association with memory scores were identified in svMCI patients (Ding et al., 2015). Given all this evidence, it is much more reasonable to suggest that the fALFF of PCC. R might be associated with the damaging effects of cognitive impairment in svMCI patients.

Numerous previous studies have explored the effectiveness of machine learning in distinguishing NC from patients with SVaD, svMCI, AD, and MCI using multimodal MRI data, including diffusion, morphometry, functional, and combined features (Yang et al., 2018; Diciotti et al., 2015; Long et al., 2016; Guo et al., 2017). Among these, SVM with a linear kernel has been identified as the most effective classification algorithm for distinguishing AD from NC (Mao et al., 2017). Among all these studies, one study that used the Hurst exponent, ALFF, regional homogeneity, and gray matter density as classification features achieved an AUC of up to 0.97 in differentiating 29 MCI patients from 33 NC (Long et al., 2016). Additionally, two other studies confirmed that a combination of resting-state measures such as ALFF, fALFF, and RSFC improves classification accuracy (Yang et al., 2018; de Vos et al., 2018).

However, our study is the first to classify svMCI+D patients from svMCI-D patients using SVM, achieving a relatively high AUC of 0.96 with only five functional features: the mean regional fALFF of OFC. R, PCC. R, and CERE. R, as well as RSFC between PCC. R and PreCUN. R, and RSFC between CERE. R and LING.R. These features may serve as potential biomarkers for distinguishing svMCI+D patients from svMCI-D patients and could be instrumental in guiding the clinical treatment of svMCI+D patients.

Finally, two major limitations of this study need to be stressed. First, although we used GRF corrections with strict parameters (a voxel level of *p* < 0.001 and a cluster level of *p* < 0.05) to enhance statistical power, future studies with larger sample sizes are highly recommended to validate our results. Second, while we identified increased brain activity, we did not find a significant relationship with HAMD scores in svMCI+D patients. This lack of correlation may be due to the relatively small sample size, and therefore, caution is warranted in interpreting these results.

## Conclusion

In conclusion, our results showed distinct patterns of the detrimental impact of cognitive impairment and depression symptoms on spontaneous brain activity in svMCI patients. However, further research is required to directly confirm the compensatory effects of depression symptoms in svMCI patients. In addition, features derived from spontaneous brain activity hold potential as biomarkers for differentiating between svMCI+D patients, svMCI-D patients, and NC, which could be valuable in guiding clinical treatment for svMCI patients, such as transcranial magnetic stimulation.

## Data Availability

The raw data supporting the conclusions of this article can be accessed upon request from the corresponding author, provided that the privacy of the participants is protected and ethical approval from the hospital has been obtained.

## Footnotes

^1^ http://rfmri.org/dpabi
